# Genetic variation reveals large-scale population expansion and migration during the expansion of Bantu-speaking peoples

**DOI:** 10.1098/rspb.2014.1448

**Published:** 2014-10-22

**Authors:** Sen Li, Carina Schlebusch, Mattias Jakobsson

**Affiliations:** 1 Department of Evolutionary Biology, Evolutionary Biology Centre, Norbyvägen 18D, Uppsala 752 36, Sweden; 2 Science for Life Laboratory, Uppsala University, Norbyvägen 18D, Uppsala 752 36, Sweden; 3 Center for Macroecology, Evolution and Climate, Natural History Museum of Denmark, University of Copenhagen, Universitetsparken 15, Copenhagen 2100, Denmark

**Keywords:** Africa, approximate Bayesian computation, Bantu-speakers, migration, population expansion

## Abstract

The majority of sub-Saharan Africans today speak a number of closely related languages collectively referred to as ‘Bantu’ languages. The current distribution of Bantu-speaking populations has been found to largely be a consequence of the movement of people rather than a diffusion of language alone. Linguistic and single marker genetic studies have generated various hypotheses regarding the timing and the routes of the Bantu expansion, but these hypotheses have not been thoroughly investigated. In this study, we re-analysed microsatellite markers typed for large number of African populations that—owing to their fast mutation rates—capture signatures of recent population history. We confirm the spread of west African people across most of sub-Saharan Africa and estimated the expansion of Bantu-speaking groups, using a Bayesian approach, to around 5600 years ago. We tested four different divergence models for Bantu-speaking populations with a distribution comprising three geographical regions in Africa. We found that the most likely model for the movement of the eastern branch of Bantu-speakers involves migration of Bantu-speaking groups to the east followed by migration to the south. This model, however, is only marginally more likely than other models, which might indicate direct movement from the west and/or significant gene flow with the western Branch of Bantu-speakers. Our study use multi-loci genetic data to explicitly investigate the timing and mode of the Bantu expansion and it demonstrates that west African groups rapidly expanded both in numbers and over a large geographical area, affirming the fact that the Bantu expansion was one of the most dramatic demographic events in human history.

## 1. Introduction

With the end of the cold Younger Dryas period and the onset of the Holocene epoch around 10 thousand years ago (kya), the re-establishment of warm conditions led to increases in human population densities throughout the world [1,2]. The population increase coincides with the invention of agriculture, which was independently developed in several geographically dispersed regions [1]. One such region was west-central Africa where the first traces of archaeological artefacts that might be linked to farming practices started to appear around 7 kya [2]. In temperate regions, farming societies generally out-competed hunter–gatherer societies, and farming populations expanded very quickly. Within west Africa, the expansions and dispersals of farming populations had begun by approximately 5 kya [3,4]. The traces of the expanding west African farmers remains today in the distribution of languages, cultural practices and genetic variants across most sub-Saharan African populations.

The majority of sub-Saharan Africans (more than 200 million people) speak one of approximately 500 very closely related languages, even though they are distributed over an area of approximately 500 000 km^2^. These languages are collectively referred to as ‘Bantu’ languages, based on the word meaning ‘people’ [5], and Bantu languages are a subgroup of the Niger–Kordofanian linguistic division, which in turn is one of the four independent major linguistic groups in Africa. The current distribution of Bantu-speaking populations is largely a consequence of the movement of people (demic diffusion) rather than a diffusion of only language [6–9]. This expansion (commonly referred to as the ‘Bantu expansion’) is linked to the spread of agriculture and, possibly, the use of iron [2,10,11]. The Bantu expansion has been suggested to begin approximately 3–5 kya based on linguistic and archaeological inferences [3,6,12] and originated in the Cross River Valley, in the region of current eastern Nigeria and western Cameroon [7,10,13,14]. Groups that existed all over sub-Saharan Africa, before the Bantu expansions, were to a large extent replaced and/or assimilated by the Bantu-speaking groups, but some populations stayed (relatively) isolated in remote areas, such as the central African rainforest and the Kalahari Desert. Furthermore, traces of the assimilated groups can still be seen as specific characteristics for particular Bantu-speaking groups such as unique genetic variants, language characteristics and cultural practices.

**Figure 1.**
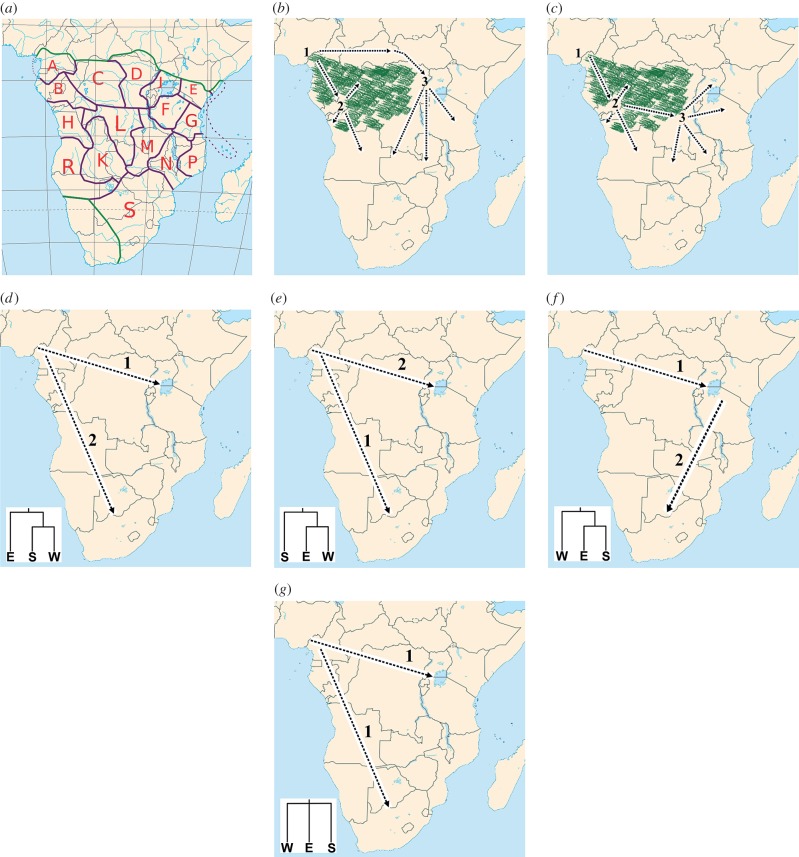
Map of sub-Saharan Africa illustrating (*a*) the different Bantu-language sub-groups according to the Guthrie classification [15], (*b*) the route of the Bantu expansions according to the ‘early-split’ linguistic model (redrawn from Pakendorf *et al*. [4]), and (*c*) according to the ‘late-split’ linguistic model (redrawn from Pakendorf *et al*. [4]). (*d*–*g*) The different models of the Bantu expansion tested in this study using an ABC approach; (*d*) the ESW model which posits a primary expansion towards the east (1) and a later expansion to the south (2), (*e*) the SEW model which posits a primary expansion to the south (1) and a later expansion to the east, (*f*) the WES model which posits a primary expansion to the east (1) and the southern expansion (2) originated from the populations that migrated to the east, and (*g*) the STAR model which posits a simulations expansion to the east and the south from the west.

Bantu languages are divided into three major groups (figure 1*a*), including northwestern Bantu (subgroups A, B and C), eastern Bantu (subgroups E, F, G, J, N, P and S) and western Bantu (subgroups H, K, L, R, D and M) [3,12,15]. Northwestern Bantu languages are spoken near and around the core region from where the expansion started; and two hypotheses have been proposed of how the eastern and western branches spread out from their west African homeland. In the first hypothesis (‘early-split’ hypothesis), the eastern and western branches split early into two separate migration routes (figure 1*b*). The ancestors of eastern Bantu-speakers are thought to have migrated directly eastwards out of the Cross River Valley, reaching the Great Lakes region in eastern Africa by approximately 3 kya [6]. Thereafter, they expanded further southwards, reaching their current distribution, across most of eastern and southern Africa, by roughly 1 kya. The ancestors of western Bantu-speakers, in turn, migrated directly south through the rainforests from the Cameroon homeland, possibly following the Atlantic coast, forming the second major route of migration [2,3,6]. The alternative hypothesis (‘late-split’ hypothesis) is that these two branches split later after the passage through the central African rainforest (figure 1*c*). A recent extensive linguistic study based on more Bantu languages with a better regional distribution used character-based Bayesian tree inference methods to reconstruct the Bantu language tree and found strong support for the ‘late-split’ hypothesis [16].

Most hypotheses about the Bantu expansion have been based on linguistics, ethnography and archaeology. More recently, genetics have also started to contribute to inferences about the Bantu expansion. Early genetic studies noted considerable genetic homogeneity among Bantu-speakers compared with the genetic differentiation between west African Niger–Kordofanian speakers and east African Nilo-Saharan speakers [17]. Studies on the single locus mitochondrial DNA (mtDNA) [18–23] and Y-chromosome markers [24–31] have shown that specific haplogroups can be associated with Bantu-speaking people. The paternal lineages of the Y-chromosome is especially useful to infer the spread of the Bantu-speaking people as, owing to patrilocality, the paternal lines are less affected by gene-flow from groups that are being displaced/absorbed in the expansion wave, than the maternal mtDNA lineages. A recent Y-chromosome study suggested multiple initial expansions of Bantu-speaking groups along the eastern and western routes and a later exclusively eastern route of expansion coupled to the invention and use of iron [30]. Other Y-chromosome studies also mentioned a likely complex process giving rise to the current spread of Bantu-speaking groups [8,26,28–30]. Recently, genome-wide typing and analyses of microsatellite markers [8,32] and single nucleotide polymorphisms [9] demonstrated the genetic similarity of geographically distant Bantu-speaking groups. De Filippo *et al.* [8] used a combined linguistic and genetic approach to test the ‘late-split’ and ‘early-split’ hypotheses and found that the late-split linguistic hypothesis fits the genetic data better, thus suggesting a more recent development of eastern Bantu languages out of western Bantu languages.

For southern Africa, there are two main Bantu-speaking groups: southeastern (subgroup S) and southwestern (subgroup R and K) Bantu-speakers. According to the linguistic hypotheses, the southwestern Bantu-speakers migrated from west Africa along the western coast and through central Africa, whereas the southeastern Bantu-speakers migrated from east Africa [2,3,6,15]. When the Cape of Good Hope was colonized by Europeans during the 1600s, the eastern branch of Bantu-speakers (specifically the Xhosa speakers) reached as far south as the Fish River in the present eastern Cape province of South Africa. Generally, the whole eastern part of the present South Africa was occupied by the southeastern branch (subgroup S) of Bantu-speakers, whereas the western parts of South Africa and the south and central parts of Namibia was occupied by Khoe herders (speaking a Khoisan click-language, unrelated to Niger–Kordofanian languages). The western branch of Bantu-speakers (subgroup R) had then just reached the north of Namibia where their spread further south was halted by the Khoe herders [6]. However, the genetic relationship among today's (geographically) west, east and southern African Bantu-speakers has not been thoroughly investigated to decipher the larger scale population movements during the Bantu expansion.

In this study, we investigate the genetic signal of the Bantu expansion across a large panel of sub-Saharan populations. We investigate the patterns of variation in a large number of microsatellites typed by Tishkoff *et al.* [32]. As the mutation rate of microsatellites is high (compared with most other types of polymorphism data), they can be particularly informative about recent demographic events. We perform a supervised clustering analysis to confirm that the Bantu expansion to a large extent involved the expansion of people and we visualize the spread of the west African genetic component across the African continent. Using an approximate Bayesian computation (ABC) approach, we estimate the timing of the Bantu expansion and contrast four different population histories related to possible routes of dispersal of the eastern branch of Bantu-speakers on the African continent.

## 2. Material and methods

### (a) Dataset description

In this study, we re-examine the microsatellite data from Tishkoff *et al*. [32]. Microsatellite data have the ability to capture information of recent demographic events owing to their particularly high mutation rate, on the order of about 10^−4^, [33,34], which result in a large number of variants that have emerged from recent mutation events. The dataset was filtered for 50% marker missingness in African populations and all indels were removed. Filtered data comprised the same 717 microsatellites for all individuals.

### (b) Supervised Structure analysis

The individuals' genomes were assigned to pre-defined and/or undefined clusters based on the microsatellite genotype data using a supervised clustering algorithm implemented in Structure v. 2.3.2.1 [35]. With the supervised Structure analysis, we aimed at determining and visualizing the spread of the west African genetic component in various groups across the African continent. Three clusters were pre-defined to contain individuals from Europe, the Middle East and South Asia, and west Africa respectively; see the electronic supplementary material, table S1. The west African group was restricted to Niger–Kordofanian individuals from Nigeria and Cameroon. Pygmy groups were not included in the fixed west African cluster and owing to the previously reported high proportion of European/Middle Eastern ancestry in the nomadic Fulani groups [32], these groups were also not included in the pre-defined west African group. The European and Middle Eastern/South Asian pre-defined clusters were used to detect recently admixed African individuals.

For the Structure analyses, we used the admixture model, using the F model of correlated allele frequencies across clusters. Each replicate Structure run used a burn-in period of 20 000 iterations, followed by 20 000 iterations from which estimates were obtained. We replicated the Structure analysis 10 times for each number of assumed clusters (*K*), from *K* = 4 to 10. The 10 replicates for each choice of *K* were summarized with Clumpp v. 1.1.1 [36] to identify common modes among replicates. The Clumpp analysis used the LargeKGreedy algorithm with 10 000 random permutations. Common solutions were identified by the Clumpp pairwise *G*′ values. All pairs with a symmetric similarity coefficient *G*′ > 0.9 were selected to be representative of a single mode. For each *K*, we used the most frequently occurring mode identified and ran Clumpp a second time (using the LargeKGreedy algorithm and 10 000 random permutations), using only the replicates belonging to this mode. From the second analysis, we obtained the mean across replicates of the cluster membership coefficients of each individual, for each mode at each value of *K*. The clustering results were visualized with Distruct [37]. We further visualized the distribution of the ancestry fraction of the pre-defined west African cluster on a map for the whole African continent through a Kriging procedure and heat plot in R (using the ‘fields’ library [38]).

### (c) Inferring the expansion characteristics of west African populations

We extracted populations that belong to the Niger–Kordofanian linguistic grouping (denoted as the NK group) from the Tishkoff *et al.* [32] data. The extracted NK group comprised 940 individuals. A second group was also extracted, which was a subset of the NK group and included 661 individuals from populations classified as Bantu-speakers (denoted as the BS group). Pygmy and Fulani groups were not included in the NK and/or BS groups.

We first investigated potential population expansion using a single-population model for both the NK group and BS group. We assumed a model of population expansion (exponential growth) starting at time *T*_EXP_ (backwards in time; see the electronic supplementary material, figure S1). An ABC [39] approach (with local linear regression adjustment) was used to estimate the expansion time *T*_EXP_ and the past population size *N*_p_ of the two groups.

To simulate population genetic data that mimics the empirical microsatellite data, we used Hudson's ms program [40] and we converted the binary output of ms to microsatellite data based on a stepwise mutation model. Specifically, we used a symmetric generalized stepwise mutation model to generate simulated microsatellite data [41–44]. Changes of the number of repeats in each mutation event followed a geometric distribution with parameter 0.95. The mutation rate *μ* of each locus was assumed to be random draw from a uniform distribution in [0.00025, 0.00075] per locus per generation. All microsatellite loci were assumed to be independent (i.e. unlinked). Electronic supplementary material, table S2, gives the parameter settings of the ABC approach. Recent population sizes are particularly difficult to infer from genetic variation [45] and we therefore chose to treat the current population size as a nuisance parameter. We investigated several choices of priors for the current population size (including one order of magnitude larger or smaller) and found that the choice had little impact on the posteriors for the parameters of interest. The summary statistics used for the ABC approach in this analysis were: (i) expected heterozygosity, (ii) variance of the number of repeats, (iii) number of alleles [46], (iv) frequency of the most frequent allele, and (v) number of singletons. For each summary statistic, we computed the mean and variance across all loci of each group (BS or NK). Times in generations were converted to times in years using 25 years per generation in all analyses.

### (d) Testing the connection among west African, east African and southern African Bantu-speakers

**Figure 2.**
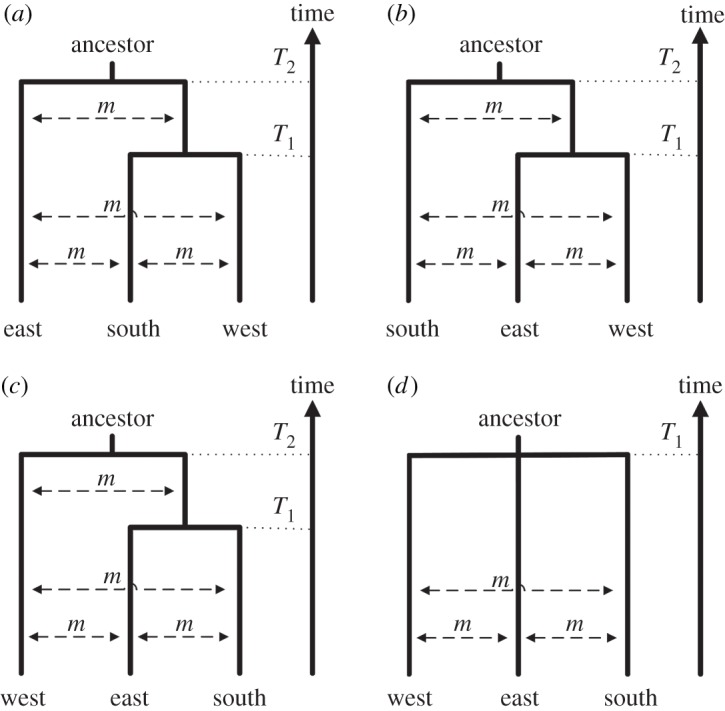
Population topology of four investigated models: (*a*) the ESW model where the population topology is (east, (south, west)), (*b*) the SEW model where the population topology is (south, (east, west)), (*c*) the WES model where the population topology is (west, (east, south)), and (*d*) the STAR model where all three groups have a common split time.

In a second analysis, we tested four different divergence models for six Bantu-speaking populations with a distribution comprising three geographical regions in Africa: eastern Bantu-speakers (Pare from Tanzania and Luhya from Kenya, sample size 40), southern Bantu-speakers (Xhosa and Venda from South Africa, sample size 41) and western Bantu-speakers (Bulu and Lemande from Cameroon, sample size 48). Figure 2 shows the population topologies of the four tested scenarios. In model ESW, the eastern Bantu-speakers split off at *T*_2_ from the ancestral population of the southern and the western Bantu-speakers, who later diverge at time *T*_1_. In other words, the southern and western Bantu-speakers share a more recent ancestry compared with eastern Bantu-speakers, which would be expected if the migration of Bantu-speaking groups to southern Africa was instigated more recently in time compared with the migration of Bantu-speaking groups to eastern Africa (figure 1*d*). In model SEW, the eastern and western Bantu-speakers share a more recent ancestry than with the southern Bantu-speakers (figure 1*e*) and in model WES, the eastern and southern Bantu-speakers share a more recent ancestry (figure 1*f*). For the three models above, we assume that migration occurs between each pair of populations with rate 4*N*_0_*m* = 1500, where *N*_0_ is the population size at present (note that since *N_e_* decrease backwards in time, the fraction of the population that is made up of migrants stays constant, *m*, but the number of migrants (2N_e_*m) decrease backwards in time). In the last model, the STAR model, all three populations diverged at the same time, *T*_1_ (figure 1*g*). Population growth (with rate *α*) is allowed in the models for each non-ancestral population, for instance for model ESW, the eastern Bantu-speaking population can start to grow at time *T*_2_, and the southern and western Bantu-speaking groups can start to grow at time *T*_1_. The ancestral populations were modelled as constant-size populations. The electronic supplementary material, table S3, gives the parameter setting of the ABC approach for this investigation of which population topology fits the genetic data best (current population sizes were not inferred). For this investigation, we used the same five summary statistics as above in addition to the three pairwise *F*_ST_s [47].

The ABC approach used 100 000 replicate simulations of sets of 717 microsatellite loci. We used 10 summary statistics (mean and variance for five summary statistics) for the population expansion investigation and 18 summary statistics (mean and variance for five within-population summary statistics of each population and mean for three between-population summary statistics (*F*_ST_)) for the population topology investigation to capture the properties of the population genetic data. The Euclidean distance between each simulated dataset and the real data was computed to obtain the approximate likelihood of the data given the particular draw of parameters from the prior distributions. The rejection tolerance was set to 0.3%, which means that the 300 simulated datasets with the shortest Euclidean distance to the real data were accepted. To obtain the posterior distribution, we transformed the summary statistics [48] followed by a local linear regression adjustment of the accepted candidate parameters [39]. To make sure that the estimated models were reasonable, we performed posterior predictive checks [49] by simulating 10 000 replicate datasets using the parameters of the estimated models (the parameters were drawn from their posterior distributions) and compute the set of summary statistics. We used principal component analysis to summarize the summary statistics computed from these simulations of the estimated model into two dimensions [50–52].

## 3. Results

**Figure 3.**
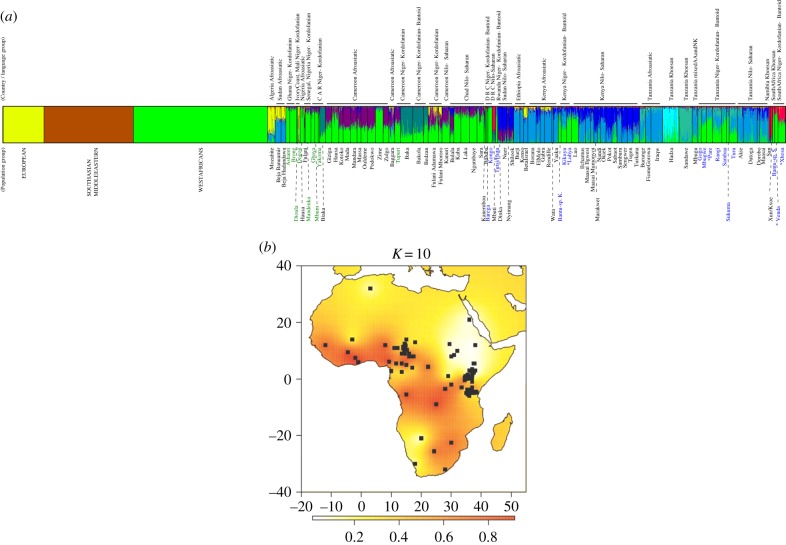
Distribution of the west African genetic component across the African continent: (*a*) supervised Structure analysis to show the distribution of the west African component (fixed green cluster), in the rest of Africa. Two other fixed clusters are European (yellow) and Middle Eastern/South Asian (brown) to account for non-African admixture into African groups. In total, 10 clusters were assumed (seven free assignments allowed). Increasing the number of clusters, *K*, from 4 (one free assignment allowed) to 10 (seven free assignments allowed) are shown in the electronic supplementary material, figure S2. Populations in coloured text were used when testing the expansion model using ABC approaches; populations in blue text are Bantu-speakers that were included in the ‘BS’ group during ABC analysis; while populations in green text are Niger–Kordofanian speakers that were included in the ‘NK’ group together with the ‘BS’ populations. Stars indicate populations from east and southern Africa that were used in the ABC analysis which tested different divergence models. (*b*) Heat map of the west African genetic component on the African continent at *K* = 10 (electronic supplementary material, figure S3 contains additional heat maps of the west African component with increasing number of clusters allowed in the supervised Structure analysis).

We interrogate genetic data to better understand the spread of the west African genetic component that accompanied the expanding Bantu-speaking people, from the region that the Bantu expansion is postulated to have started from (Nigeria and Cameroon), throughout the rest of the African continent. In a supervised clustering analysis, the west African ancestry was clearly visible throughout the whole of sub-Saharan Africa (light green component in figure 3*a* and dark red component in figure 3*b*). A reduction in the west African component is seen for the regions where other separate linguistic groups still coexist with Niger–Kordofanian/Bantu-speaking groups (Afro-Asiatic in northern Africa; Nilo-Saharan, Afro-Asiatic and Khoisan for eastern Africa; and Khoisan for southern Africa). The distinct clusters for these three different additional African linguistic groups also became apparent as the number of assumed clusters (*K*) increased (figure 3*b* and electronic supplementary material, S2; see also [9,32]) but the west African genetic component remains present in many populations and areas of the African continent (figure 3; electronic supplementary material, S2 and S3).

### (a) Inferring the onset of population expansion

To further investigate the demographic parameters of the Bantu expansion, we used an ABC approach to estimate the timeframe and route of the expanding west African Bantu-speakers. We use the west African Niger–Kordofanian group as comparison for the general demographic changes in west Africa.

**Figure 4.**
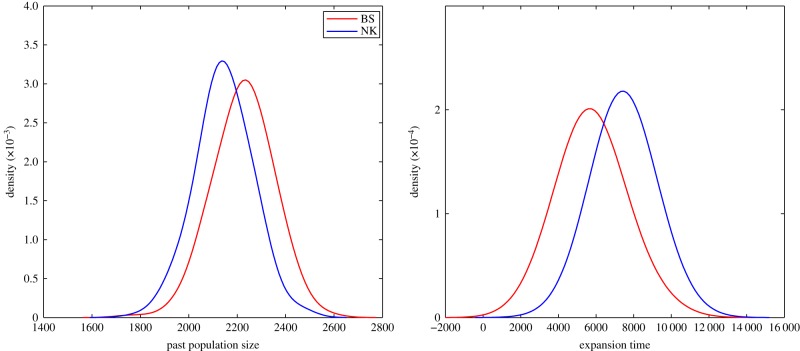
The posterior distribution of (*a*) the past population size *N*_p_ and (*b*) expansion time *T*_EXP_ and for the Bantu-speaking group (red) and the Niger–Kordofanian-speaking group (blue) group.

Figure 4 and table 1 show the estimation of the expansion time and the past population size for the NK and BS groups. For both the NK and BS groups, we estimate a relatively recent population expansion, but the start of expansion of the BS group was more recent (about 5600 years ago) than for the NK group (about 7400 years ago). The past population size of the BS group and the NK group were estimated to be very similar (and relatively small, about 2200 and 2100, respectively), but note that these estimates critically depend on assumptions about the mutation rate.

**Table 1.** Estimated past population size (mean and 95% confidence interval in brackets) in the Bantu-speaking group and the Niger–Kordofanian-speaking group.

	past population size	expansion time
Bantu-speaking	2230 [1967, 2454]	5646 [3202, 8871]
Niger–Kordofanian	2147 [1918, 2355]	7399 [5765, 9616]

To make sure that the estimated models were reasonable, we performed posterior predictive checks [49] by simulating 10 000 replicate datasets using the parameters of the estimated models (the parameters were drawn from their posterior distributions), compute the set of summary statistics and compare to the empirically observed set of summary statistics. For the BS and the NK groups, the summary statistics of the empirical data falls within the 95% envelopes of the summary statistics simulated from the posteriors (see the electronic supplementary material, figure S4). In summary, single population models of population growth can capture some important features of the underlying demographic scenario, but there are clearly additional factors that can contribute to the empirical patterns of genetic variation that are not captured by single population models, such as the assimilation of other peoples and migration from other groups.

### (b) Inferring the scenario of expansion of west Africans during the Bantu expansion

**Figure 5.**
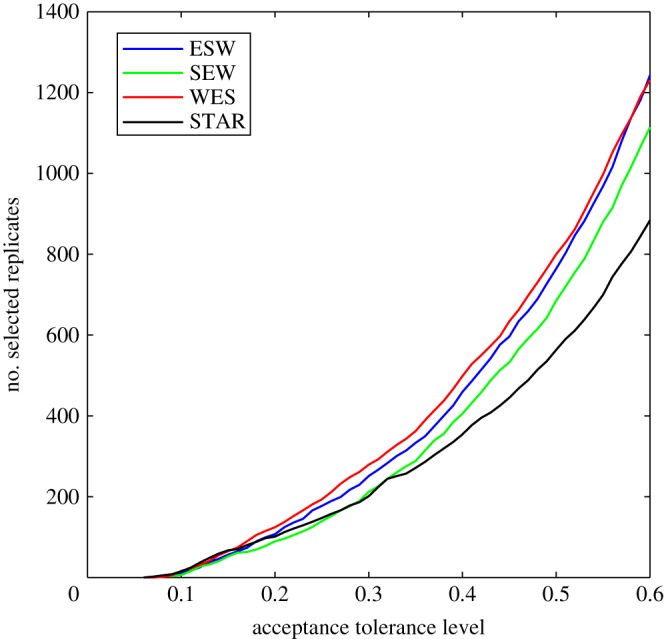
The number of accepted simulated replicates as a function of a fixed tolerance value for all four models.

We investigated four different models describing the population history of Bantu-speaking groups from west, east and south Africa. In order to determine which model has the greatest statistical support, we plot the fraction of accepted simulations for each model as a function of a fixed tolerance value (figure 5). For basically the entire range of tolerance values, the WES model received the greatest support (the ratio of accepted simulations for two models is an approximation of Bayes factors, which are, for the WES model versus ESW, SEW and STAR models 1.11, 1.28 and 1.30, respectively). Hence, there is only weak support of the WES model, in particular, compared with the ESW model. More importantly, all models give relatively similar estimates of the divergence times; the first (backwards in time) split (*T*_1_) around 4000–5000 years ago and the second split (Δ*T* = *T*_2_ − *T*_1_; except the STAR model) about 1000–2000 years earlier (electronic supplementary material, table S4). The posterior predictive check for the WES model demonstrates that the inference from this model is robust (electronic supplementary material, figure S5) in that the WES model can produce patterns of genetic variation that mimic the empirical patterns.

## 4. Discussion

It is well known that Bantu languages are spread throughout sub-Saharan Africa but trace their origin to west Africa. Various linguistic studies have contributed towards resolving the Bantu language trees and helped to infer the proposed routes of the expansion of Bantu-speaking people [3,6,12,15]. Although linguistic studies provide a valuable resource in predicting past population movements, it is merely indirect evidence of migration and it is not a given that the spread of languages is accompanied by genes and people. Linguists have warned against such assumptions and it is well known that whole population language shifts can occur [3]. Although a cultural diffusion and language shift scenarios involving Bantu-speakers was proposed by some genetic studies [53], most single marker and autosomal genetic studies supports a major demic diffusion for Bantu-speakers with notable but low amounts of gene-flow from resident populations. Our study supports this observation of a primarily demic diffusion of Bantu-speaking people from west Africa and clearly visualizes the spread of the west African genetic component throughout sub-Saharan Africa.

We also dated the start of expansion of west Africans using an ABC approach applied to both Niger Kordofanian speakers and a subset of that group; Bantu-speakers. The analysis showed that the expansion of the BS group was more recent (about 5600 years ago) than for the NK group (about 7400 years ago). We note that these expansion time estimates may be downwardly biased as both the NK and the BS groups contain pooled samples from several populations [54]. However, the pooled populations show very little evidence of population structure and our aim was to compare the relative expansion times for the two groups rather than the absolute values. An expansion in the Niger–Kordofanian linguistic phylum has been tentatively linked with the improving Holocene climate (12–10 kya) [55]. In a previous genetic study of multilocus autosomal re-sequencing data from the west African (Niger–Kordofanian speaking) Yoruba and Mandenka populations, Cox *et al*. [56] used a two-phase growth model and found a sevenfold population expansion around 31 kya (assuming 20 years per generation). However, they could not reject the possibility of an expansion around the start of the Holocene for these farming populations, whereas for the San hunter–gatherer population, population growth during the Holocene was rejected [56]. The authors however acknowledged that the limited size of their dataset had more power to infer older rather than more recent growth [56]. Analyses of the current dataset date the expansion of Niger–Kordofanian groups to more recent times. The estimated onset of expansion of the NK group (7400 years ago) may reflect the start of (perhaps more rapid) population growth in west African populations and coincides with an appearance in the archaeological record of artefacts (pottery, ground-stone and hoe-like instruments), which might be the first indications of farming in west Africa [2]. Furthermore, it is around this time that populations in western Africa adopted a more settled lifestyle [2].

Our estimates of an expansion event in Bantu-speakers postdate the expansion in the NK group by approximately 2000 years. This genetic-based dating of the start of the expansion of Bantu-speaking people (5600 years ago) corresponds well with a combined archaeological and linguistic estimate of the start of the Bantu expansion [12]. Holden *et al.* [12] used maximum-parsimony methods to infer a Bantu language tree that reflects the spread of farming across sub-Saharan Africa to between approximately 5000 and 2500 years ago. In the language tree, modern Bantu language subgroups, defined by clades on the tree, mirror the earliest archaeological farming traditions both geographically and temporally [12].

Both linguistic [12,16] and genetic studies [8] previously tested models that dealt with the routes of spread of Bantu languages. Linguistic models supports two migration routes, an eastern and a western route, in which Bantu languages are thought to have spread to the east and the south of Africa. There are two hypotheses regarding the time of association of the eastern and western branches before they split into two, namely, the ‘early-split’ and ‘late-split’ hypothesis. These models mainly propose longer/shorter associations of eastern and western Bantu languages (figure 1*b*,*c*). Considering the eastern migration route alone, two alternative routes around the central African rainforest towards the east of Africa have thus been proposed by linguists, and genetic studies tested these two hypothesis and found more support for the ‘late-split’ hypothesis [8]. The model we tested is different from the ‘late-split’ and ‘early-split’ hypotheses and relates to the subsequent spread of the eastern branch of Bantu-speakers to the south of Africa (cf. figure 1*b*,*c* versus 1*d–g*).

Our investigation of different population histories among (geographically) west, east and southern African Bantu-speakers showed that the WES model describes the data the best. Thus, the movement of southeast Bantu-speakers (such as the Xhosa and Venda) to the south of Africa was inferred to follow a path via eastern Africa. This finding fits well with the linguistic model, in which speakers of ‘southeastern’ Bantu languages (subgroup S in linguistic terms) are related to or descendent from east African Bantu languages [3,6,12,15,16]. Note, however, that the WES model is only marginally better supported compared with the ESW model. Furthermore, only the eastern route of the Bantu expansion was tested in this study. Linguistic studies propose that western Bantu-speakers spread directly south from Cameroon, forming a second major route of migration to the south. As no southwestern Bantu-speakers (subgroup R and K) were included in the Tishkoff *et al.* [32] dataset, potential migration along the western route could not be investigated.

It has been suggested that the southeastern and southwestern Bantu-speaking groups mixed after the initial split based on overlapping occupation in the (present day) region of southern Zambia [57]. This subsequent contact between the eastern and western streams might explain the fact that the ESW model received the second greatest support in our ABC analysis—as a consequence of southeastern Bantu-speakers receiving genetic material from southwestern Bantu-speakers. Future investigations that include southwestern and central African Bantu-speakers may aid in refining our understanding of the large-scale spread of Bantu-speakers.

There is a clear signal of admixture from resident population groups in the south (Khoisan-speakers) and in the east (Nilo-Saharan and Afro-Asiatic speakers). Admixture could potentially affect the population history inference, but it should only impact the results if there was admixture from a particular group into more than one Bantu-speaking group. The admixture in eastern and southern Bantu-speakers originates from indigenous and distinct populations [9,32] and it is unlikely to impact the general inferred population history of the (geographically) west, east and south Bantu-speakers.

## 5. Conclusion

We investigated various aspects of the Bantu expansions using genome-wide microsatellite markers and confirm the spread of a west African genetic component across the whole of sub-Saharan Africa. We found that the Bantu expansion occurred later than general expansions within peoples living in west Africa. Our study furthermore investigated the modes of the large-scale movements, of Bantu-speaking people within Africa and found that the most likely genetic model for spread of the eastern branch of Bantu-speakers is a spread of people to the east followed by a spread of people to the south. Our study represents, to our knowledge, the first genetic study that tests the mode of spread of eastern Bantu-speakers to the south of Africa. Further analysis that includes southwestern and central African Bantu-speakers can refine and extend hypotheses regarding other large-scale movements of Bantu-speakers and models that include admixture from resident groups will probably improve the resolution.

## Supplementary Material

Supplementary Material
