# Supplementary material for: Genetic variation reveals large-scale population expansion and migration during the expansion of Bantu-speaking peoples
Source: Proc Biol Sci. 2014 Oct 22;281(1793):20141448. doi: 10.1098/rspb.2014.1448 (PMC4173682; doi:10.1098/rspb.2014.1448)
Supplement: Supplementary Material [file rspb20141448supp1.pdf]

Supplement Li et al

Supplementary Tables and Figures

Table S1. Populations included in fixed clusters during supervised STRUCTURE analyses

Cluster	Populations in fixed cluster
1	Sardinian, Italian, Basque, Russian, Adygei, Orcadian, French
2	Bedouin, Druze, Palestinian, Brahui, Balochi, Makrani, Sindhi, Pathan, Kalash, Burusho, Hazara, Temani
3	Mvae, Ngumba, Bamoun, Banen, Bafia, Lemande, Batanga, Yoruba(CEPH), Bassange, Yoruba, Igbo, Igala, Gwari, Banen, Mvae, Iyassa, Fang, Mabea, Yambassa, SouthTikar, NorthTikar, Ntumu, Bulu, Fang, Ntumu, Gwari, Wimbum, Batie, Ewondo, Eton, Koma

Table S2. The prior parameter settings for the ABC analyzes of single-population models. The prior distributions of the past population size (Np) and the start-time of expansion (T_{EXP}) are uniform. The present population size (Npr) was assumed to be uniform in logarithmic scales (corresponding [1.0×10^6, 5.0×10^6]).

	BS group	NK group
**$\log_{10}(N_{pr})$**	[6, 6.698]	[6, 6.698]
**N_p**	[100, 10 000]	[100, 10 000]
**T_{EXP} (years)**	[100, 40 000]	[100, 40 000]

Table S3. The prior parameters for the four divergence models. The present population sizes for the east, west and south Bantu-speaking groups ($N_{pr,E}$, $N_{pr,W}$, and $N_{pr,S}$) were set to be uniform distribution [5, 6] in logarithmic scale (corresponding [1.0×10^5 1.0×10^6]). The prior distributions of past population sizes ($N_{p,E}$, $N_{p,W}$, and $N_{p,S}$) and the divergence times were uniform.

Parameter	ESW, SEW, WES	STAR
**$\log_{10}(N_{pr,E})$**	[5, 6]	[5, 6]
**$\log_{10}(N_{pr,S})$**	[5, 6]	[5, 6]
**$\log_{10}(N_{pr,W})$**	[5, 6]	[5, 6]
**$N_{p,E}$**	[100, 10 000]	[100, 10 000]
**$N_{p,S}$**	[100, 10 000]	[100, 10 000]
**$N_{p,W}$**	[100, 10 000]	[100, 10 000]
**T_1 (years)**	[100, 20 000]	[100, 40 000]
**$\Delta T = T_2 - T_1$ (years)**	[0, 20 000]	-

Table S4. Estimation of population sizes and divergence times for the four population models.

		ESW	SEW	WES	STAR
**N_E**	**Mean**	330	1000	1457	812
	**95% CI**	[124, 991]	[227, 2556]	[295, 3656]	[189, 2257]
**N_S**	**Mean**	827	406	471	447
	**95% CI**	[211, 2210]	[126, 1206]	[163, 1024]	[173, 1337]
**N_W**	**Mean**	1042	824	671	545
	**95% CI**	[225, 2368]	[234, 2034]	[147, 2061]	[150, 1391]
**T_1**	**Mean**	4038	5142	4879	4754
	**95% CI**	[2011, 12 380]	[2073, 17 274]	[2050, 14 004]	[2011, 10 434]
**ΔT**	**Mean**	1947	986	1028	
	**95% CI**	[210, 11 944]	[67, 8498]	[132, 8049]	

Supplementary Figures:

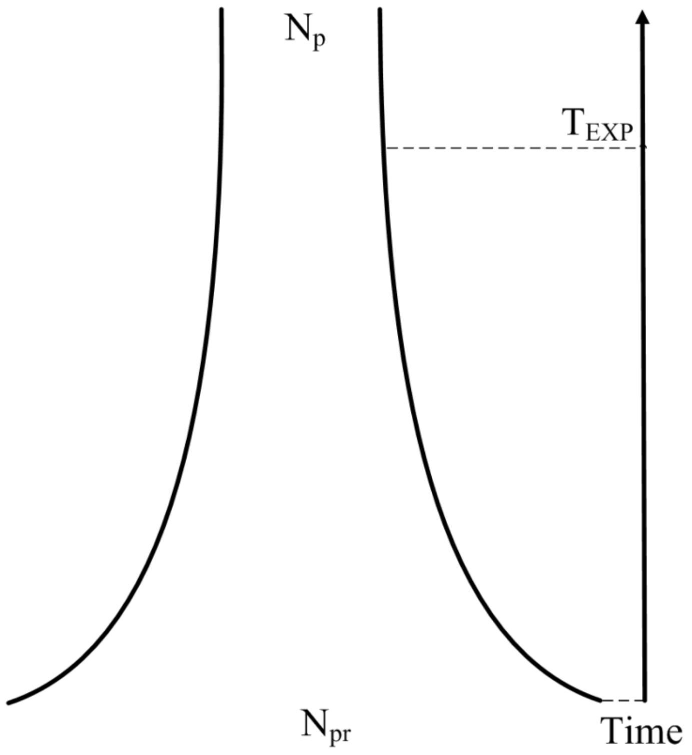

Figure S1. Single population expansion model. N_p denotes the effective population size in the past, at the start of the expansion, N_{pr} denotes the effective population size at present, and T_{EXP} denotes the time of the start of expansion.

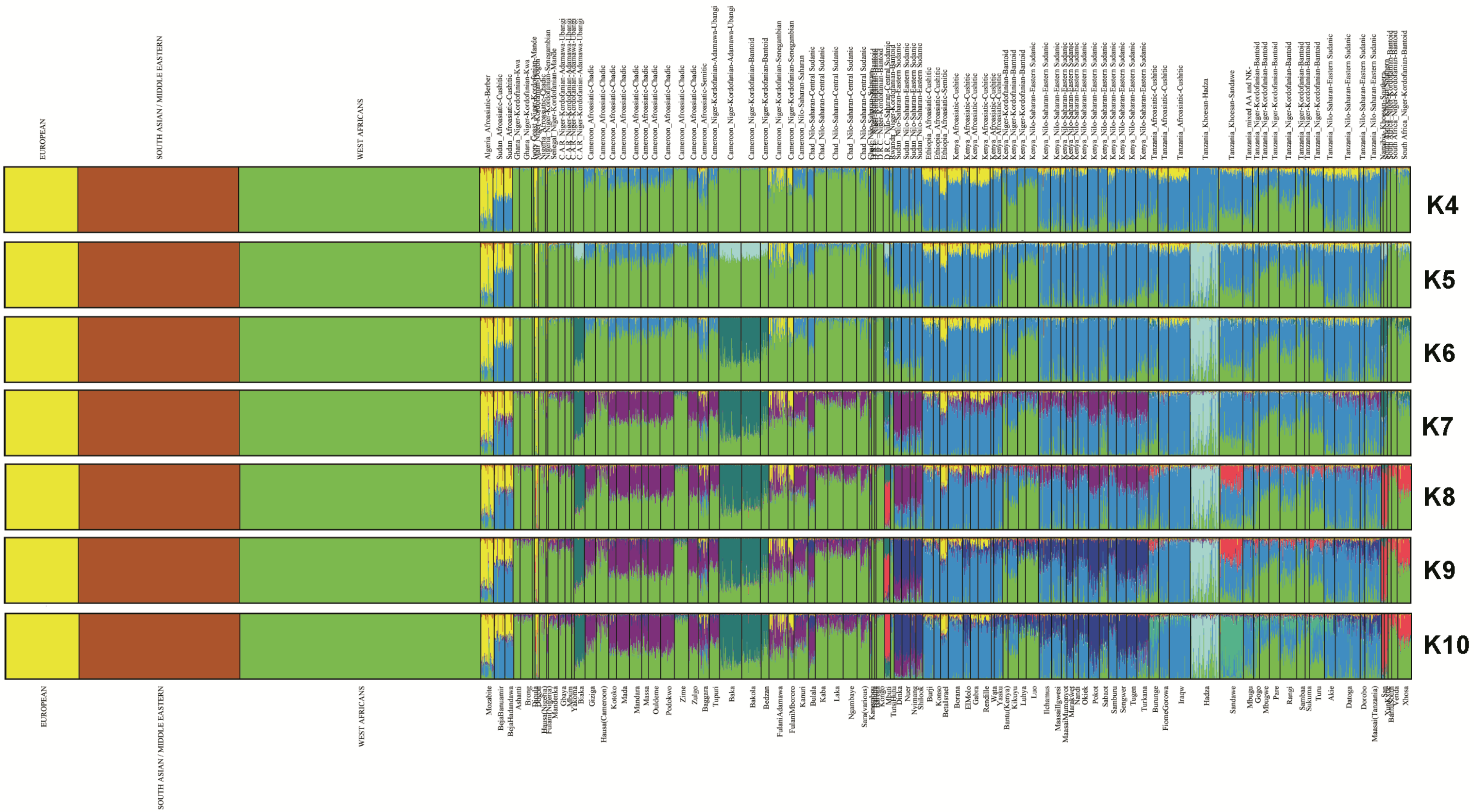

Figure S2. Supervised STRUCTURE analysis to show the distribution of the West African component (Fixed green cluster), in the rest of Africa. Two other fixed clusters are European (yellow) and Middle Eastern/South Asian (brown) to account for non-African admixture into African groups. In total, a maximum of 10 clusters were assumed (7 free assignments allowed). Increasing the number of clusters, K, from 4 (1 free assignment allowed) to 10 (7 free assignments allowed) are shown here.

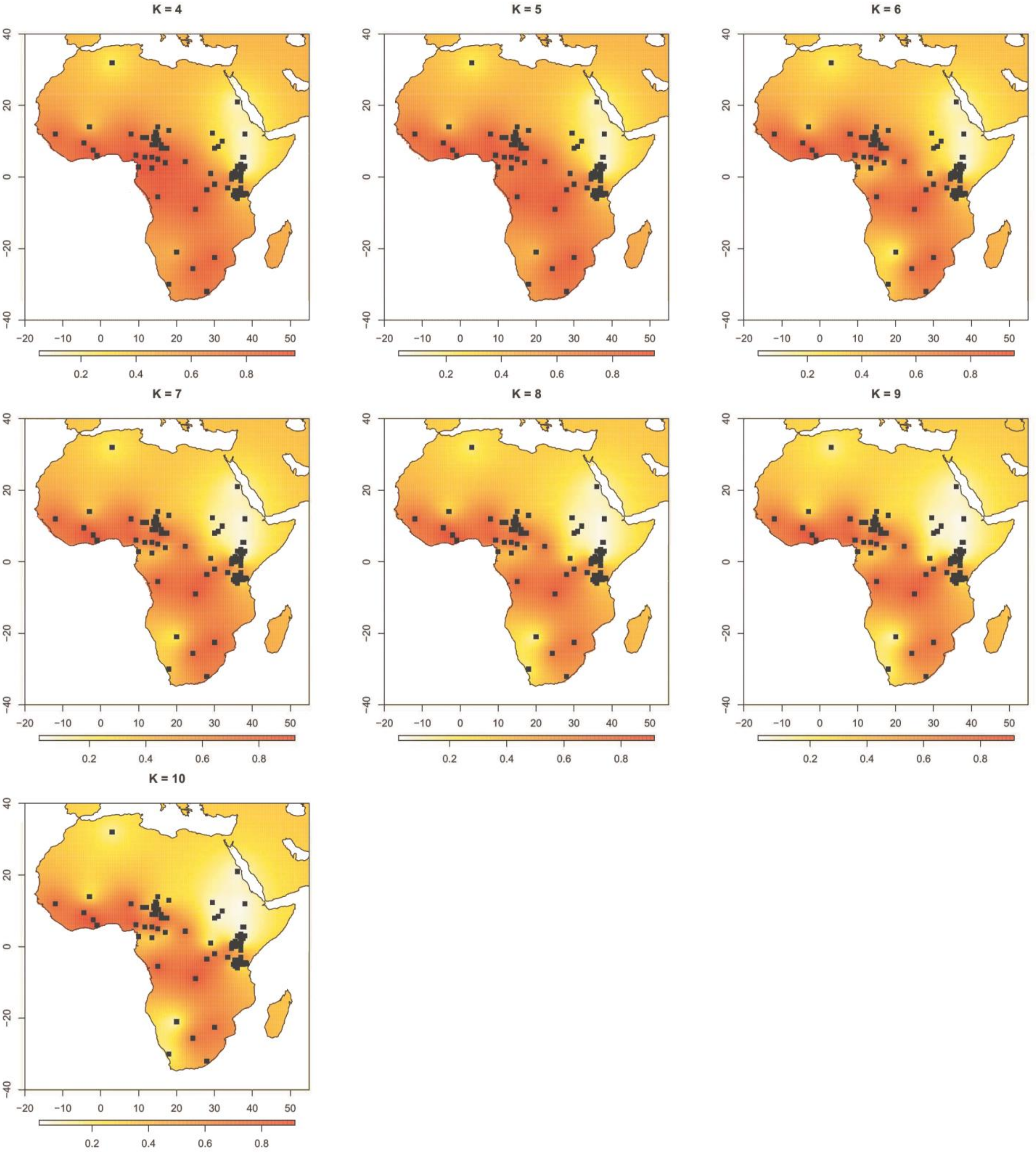

Figure S3. Heat maps of the West African genetic component on the African continent. Panels show the presence of the West-African component with increasing number of clusters allowed in the supervised STRUCTURE analysis with West African individuals (Table S1) pre-defined into a fixed cluster.

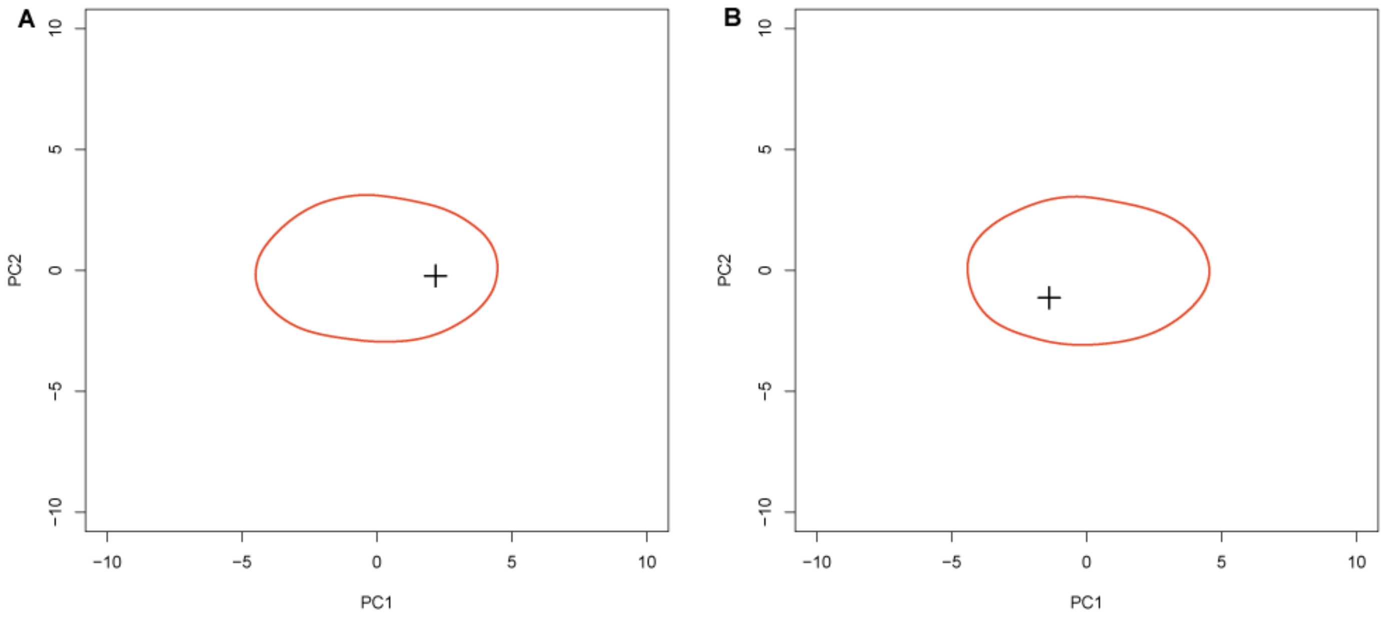

Figure S4. Posterior predictive checks for the estimation of the past population size and time of expansion based on a single-population model. The red line corresponds the 95% envelops of the posterior simulation and the crosses corresponds to the summary statistics of the empirical data. A) Bantu-speaking group, B) Niger-Kordofanian-speaking group.

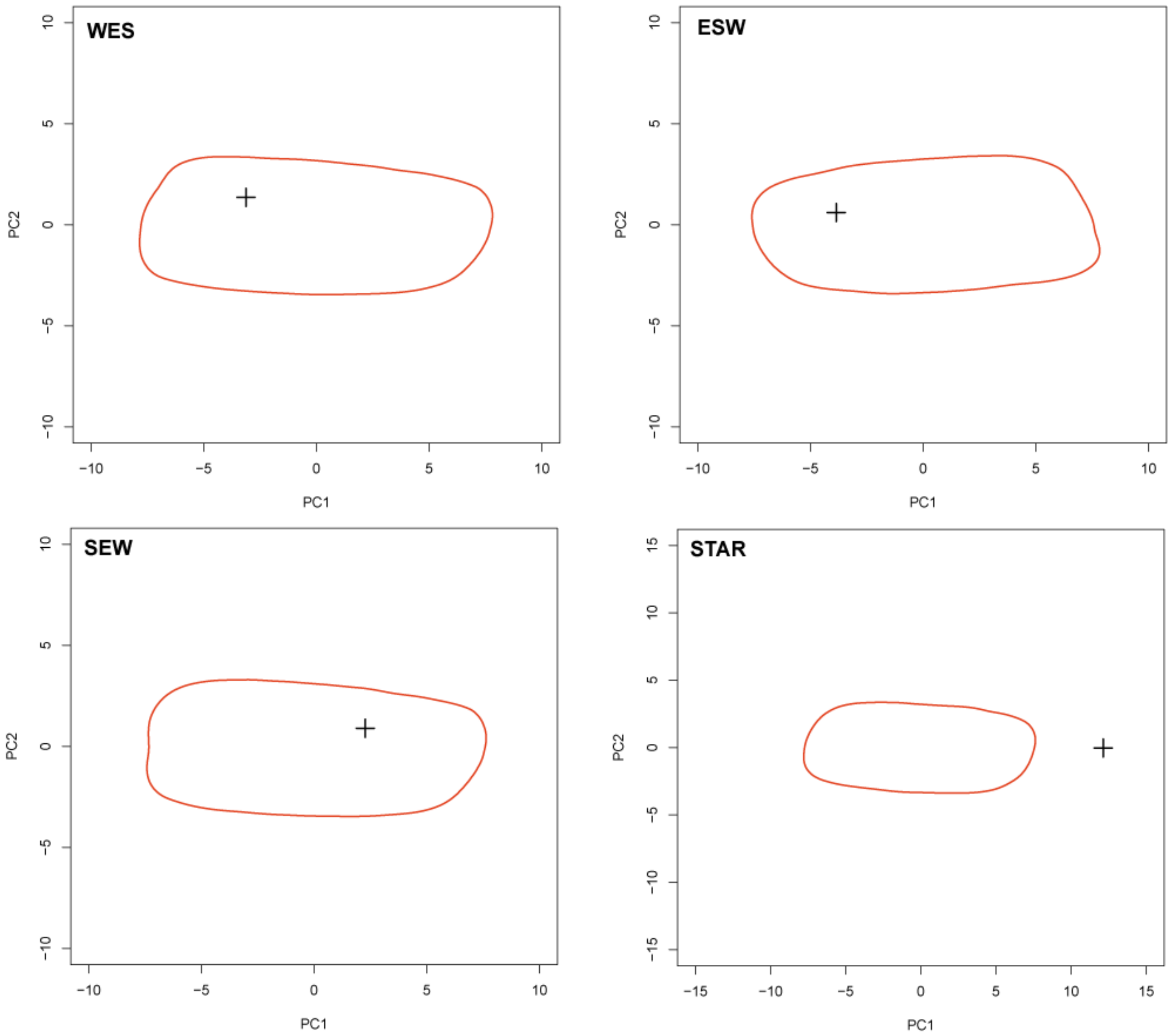

Figure S5. Posterior predictive check of the WES model. The cross marks the summary of the empirical data and the red contour gives the 95% envelop of values from the inferred model.
